# Medical Colleges

**Published:** 1874-07-01

**Authors:** 


MEDICAL COLLEGES.—The following resolutions were adopted by a full meeting of the Esculapian Society, at Marshall, May 27, 1874:

*Resolved*, That this Society hails with pride and admiration the Medical Educational Institutions of our great State of Illinois.

*Resolved*, That while we entertain the most catholic views toward medical colleges of sister States, we know of none possessing superior facilities in the West, both in regard to clinical instruction and able medical teachers.

*Resolved*, That jealousy and rivalry are as reprehensible in medical schools as in individuals, and that we will discountenance and frown down any attempt on the part of one school to supercede a sister school in any other way than by genuine merit. That we believe that the growing interests of the State require and will sustain two medical schools in Chicago, and that we can conscientiously commend the Rush Medical College and the Chicago Medical College to such as desire a thorough medical education.

---

EXTERNAL TREATMENT OF VARICOSE VEINS.—Dr. Linon says, in the *Tribune Medicale*, that he has for years treated such cases with success by swathing the leg in a flannel compress wet with a solution of chloride of iron in water, forty-five grains to the ounce, and then applying a roller flannel bandage over it firmly for twenty-four hours. This is to be repeated daily for a week or two weeks, when the patient is, or ought to be, well.—*Med. Press and Circular*.

